# Fatty Acid Regulation of Voltage- and Ligand-Gated Ion Channel Function

**DOI:** 10.3389/fphys.2016.00573

**Published:** 2016-11-28

**Authors:** Silvia S. Antollini, Francisco J. Barrantes

**Affiliations:** ^1^Instituto de Investigaciones Bioquímicas de Bahía Blanca (CONICET-UNS)Bahía Blanca, Argentina; ^2^Departamento de Biología, Bioquímica y Farmacia, Universidad Nacional del SurBahía Blanca, Argentina; ^3^Laboratory of Molecular Neurobiology, BIOMED UCA-CONICETBuenos Aires, Argentina

**Keywords:** ion channels, cell-surface receptors, ligand-gated channel, fatty acids, PUFA, FFA, VLCFA

## Abstract

Free fatty acids (FFA) are essential components of the cell, where they play a key role in lipid and carbohydrate metabolism, and most particularly in cell membranes, where they are central actors in shaping the physicochemical properties of the lipid bilayer and the cellular adaptation to the environment. FFA are continuously being produced and degraded, and a feedback regulatory function has been attributed to their turnover. The massive increase observed under some pathological conditions, especially in brain, has been interpreted as a protective mechanism possibly operative on ion channels, which in some cases is of stimulatory nature and in other cases inhibitory. Here we discuss the correlation between the structure of FFA and their ability to modulate protein function, evaluating the influence of saturation/unsaturation, number of double bonds, and *cis* vs. *trans* isomerism. We further focus on the mechanisms of FFA modulation operating on voltage-gated and ligand-gated ion channel function, contrasting the still conflicting evidence on direct vs. indirect mechanisms of action.

Free fatty acids (FFA) are customarily studied in relation to the physical adaptation of cells to their environment, the normal metabolism of lipids and carbohydrates (lipogenesis, lipolysis, esterification, oxidation, glycolysis, fatty acid uptake) and disease conditions, such as obesity, atherogenic dyslipidemias, type 2 diabetes, or insulin resistance (Boden, 2008). This broad palette is largely associated with the physiology and physiopathology of FFA resulting from the lipolytic breakdown of stored tissular triglycerides. Other diseases involving pathological affectation of fatty acids, albeit rare, include X-linked adrenoleukodystrophy, a progressive neurodegenerative disorder caused by the loss-of-function mutation in the ATP-binding cassette transporter subfamily D member 1 gene. The transporter mediates the transport of saturated very long-chain fatty acids (VLCFA), and the disease displays an abnormal accumulation of VLCFA (Mosser et al., 1993). In addition to these systemic and more general effects of FFA, the activity of many membrane proteins is modulated by the lipid composition of the membranes in which they are embedded and the physicochemical properties of the FFA produced locally are increasingly gaining attention in this regulatory role. FFA are normal constituents of biological membranes that are continuously being produced and catabolized in living cells. No consensus view has emerged to date to account for the mechanisms by which this type of lipids modulate membrane proteins (Ordway et al., 1991; Petrou et al., 1995); it is highly likely that they will turn out to be multifactorial in nature. Under certain stress conditions and most particularly in brain, the amount of endogenous FFA can increase dramatically, a phenomenon that has been interpreted as fulfilling a neuroprotective function (see Lauritzen et al., 2000; Blondeau et al., 2002; Buckler and Honore, 2005). Events like ischemia, hypoxia and kainate-induced epilepsy cause a decrease in the intracellular pH, augmented intracellular FFA concentration and an increase in the cytoplasmic Ca^2+^ concentration, leading to the activation of phospholipases. Lauritzen et al. (2000) showed that the PUFA linolenic acid (n-3 PUFA) prevents neuronal death in an animal model of transient global ischemia even when administered as long as 30 min after the insult. Linolenic acid also protected animals treated with kainate against seizures and hippocampal lesions. The augmentation of both the pH and cytosolic FFA concentrations causes hyperpolarization of the cell membrane and reduces the Ca^2+^ influx, thus deterring excitatory glutamatergic transmission and preventing neuronal death, pointing to the possible role of FFA as neuroprotective agents and anti-epileptic compounds (Lauritzen et al., 2000). In addition, dysfunction in fatty acid metabolism is implicated in disease conditions, such as cardiovascular disease, metabolic syndrome, type 2 diabetes, obesity, hypertension and proimflammatory conditions, as well as in several neurological diseases related to the alteration of insulin equilibrium in brain, as observed in Parkinson's disease, Alzheimer's disease and some forms of the schizophrenic spectrum disorders (Virmani et al., 2015).

It is well documented that FFA can directly or indirectly affect the activity of a variety of ion channels. The former involves the interaction between FFA and the ion channel protein or an associated site within the membrane without intervening factors or intermediaries; indirect effects involve the prior transformation of FFA to biologically active metabolites (Ordway et al., 1991), usually through signaling cascades. FFA effects on ion channels depend on their chemical structure: some ion channels are affected by both saturated and unsaturated fatty acids whereas others are only affected by unsaturated fatty acids (Ordway et al., 1991; Sumida et al., 1993; Meves, 1994). Similarly, *cis* and *trans* FFA isomers have been postulated to exert effects on some ion channels whereas only the *cis* configuration has been identified in other cases (Ordway et al., 1991; Sumida et al., 1993; Meves, 1994). An example of this occurs in the hypertriglyceridemia associated with acute pancreatitis. Hydrolysis of triglycerides by pancreatic lipase in acinar cells releases large quantities of FFA, which trigger an increase in cytosolic Ca^2+^ concentration, an effect that depends on the unsaturated/saturated FFA ratio. High concentrations of unsaturated fatty acids lead to an elevation of cytosolic Ca^2+^ and induce the expression of distinct isoforms of the enzyme phosphokinase C (PKC), the activity of which directly depends on the degree of FFA unsaturation (Chang et al., 2015).

Early fluorescence measurements undertaken by Karnovsky (1979) to study possible membrane alterations induced by FFA led to the classification of FFA into two groups: group A, containing *cis*-unsaturated FFA with a kink in the molecule (such as oleic and palmitoleic acids), and group B, comprising saturated and *trans*-unsaturated fatty acids with a linear structure (such as stearic and elaidic acids). This structural difference has important biophysical implications. Group A fatty acids disorder the membrane's interior and order the more shallow head group region, whereas members of group B do not alter the bilayer core but order the head group region. Such structure-dependent perturbations can lead to conformational changes in membrane-embedded proteins, a fact reflected in various communications reporting differences in biological activity for group A and group B FFA (Casabiell et al., 1991; Pérez et al., 1997, 2003).

Despite the plethora of studies concerning the relationship between FFA and proteins—in particular ionic channels and receptors- it has not been possible to find a common mechanism of action: whereas in some cases FFA stimulate some functional property of the protein in question, in other cases they act as inhibitors. Furthermore, in some cases the structure of the FFA is determinant (saturated vs. unsaturated; one or two vs. more double bonds; *cis* double bonds vs. *trans* double bonds) whereas in other cases the detailed chemical structure does not appear to play a key role; in some cases the FFA acts directly on the target protein whereas in others the FFA does so by activating an intracellular cascade that eventually results in protein modulation. In the following sections, examples of FFA modulation of distinct ion channels will be discussed with special emphasis on the voltage-gated ion channels and the nicotinic acetylcholine receptor (nAChR), paradigm of the rapid ligand-gated ion channels. From the point of view of the *messenger*, in this review we restrict ourselves to the analysis of the modulatory effects caused exclusively by FFA, without dwelling on the changes in the membrane microenvironment produced by the retailoring of the fatty acyl chain composition of either phospholipid or cholesterol ester molecular species. From the point of view of the *target* molecule, we also circumscribe ourselves to the ion channels, leaving aside e.g., the interesting field of G-protein-coupled receptors (GPCRs) acting as sensors of metabolic state, and activated by short-chain fatty acids, i.e., the so-called FFA receptors (Hudson et al., 2013).

## FFA effects on voltage-gated ion channels

### Potassium (K^+^) channels

K^+^ channels comprise a large superfamily of integral membrane proteins displaying diverse functions in essentially all organs of vertebrate and invertebrate organisms. Structurally, they possess several transmembrane (TM) helices spanning the lipid bilayer. Based on the combination of their structural and functional characteristics, the K^+^ channel superfamily can be divided into three main families: voltage-gated (Kv, 6 TMs), inwardly rectifying (Kir, 2 TMs), and tandem pore domain (K2P, 4 TMs) channels (Kuang et al., 2015). The modulation of these channels by FFA has been documented in numerous instances.

*Kv channels* are the third-largest class of signal transduction proteins, second only to G protein-coupled receptors (GPCRs) and protein kinases (Yu and Catterall, 2004). The pioneer work of Villarroel and Schwarz (1996) studied the effect of arachidonic acid (AA) over 12 different K^+^ channels expressed in *Xenopus laevis* oocytes. Different effects (inhibition or enhancement of the channel current) were observed for each channel; the greatest effect was observed in the rat K4.2 channel. The current reduction in the presence of AA was near 68%, with an almost total current recovery upon AA removal by washout or bovine serum albumin addition. A similar effect was observed in the presence of 5,8,11,14-eicosatetraynoic acid (ETYA), a nonhydrolizable AA analog. The authors discarded an effect mediated by (1) the metabolic processing of AA by cyclooxygenase, lipooxygenase or epoxygenase pathways, (2) an increment in intracellular Ca^2+^, or (3) activation of a PKC. By testing different FFA, they also discarded the possibility that the effect of AA on the Kv4.2 channel was mediated by an indirect mechanism related to alteration of the membrane fluidity. The authors postulated a direct mechanism mediated by the specific interaction of AA with the channel; although they could not find the binding site for AA, they suggested that the S4–S5 loop is indirectly involved. In the same work (Villarroel and Schwarz, 1996), the authors described a potentiation effect of AA on the Shaker Kv channel, the prototypic member of the family, with perturbations of the activation and inactivation kinetics, an effect that was not reproduced with ETYA. Later works continue the study of this effect. Modulation by FFA of the Shaker Kv channel shifts the voltage dependence of activation via an electrostatic mechanism that ends in channel activation (Börjesson et al., 2008, 2010; Xu et al., 2008). The observed effect was similar for six different polyunsaturated fatty acids (PUFA), whereas monounsaturated and saturated fatty acids produced no effect (Börjesson et al., 2008). The charge of the PUFA head group determines the direction of the effect, which has been referred to as the “lipoelectric mechanism” (Börjesson et al., 2008, 2010). FFA can also induce channel opening by affecting one or several of the steps/molecular rearrangements leading to channel opening. Studying point-mutated Shaker channels covering the lipophilic surfaces of the extracellular helices of S3, S4, S5, and S6 segments, Börjesson and Elinder (2011) concluded that residues in the voltage sensor domain are important for PUFA acting on the channel's voltage dependence, with high-impact residues clustered in a small region of the lipid-facing S3–S4 bend, where these authors tentatively assign the location of the PUFA site of action. Börjesson and Elinder (2011) further demonstrated that PUFA cause very different effects on different ion channels depending on the presence of charges at specific positions. A recent *in silico* study employing atomistic molecular dynamics simulations identified a similar potential PUFA site in the open channel state of *Shaker* K_V_, located at the lipid-facing side of a pocket connecting the extracellular linker between S3 and S4 helices (Yazdi et al., 2016). This study also provided an explanation for the activation caused by FFA: the selective stabilization of the open state of the Kv channel as PUFA established fewer interactions with the protein in the closed state. Interestingly, Yazdi et al. (2016) point out that PUFA induce channel opening by modulating voltage-sensitivity, thus opening a potential therapeutic avenue for the use of ketogenic diets in refractory epilepsy.

*Background K*^+^ *channels* (4 TMs and 2 pore domain, 2P) play a key role in setting the neuronal membrane potential, in tuning the duration of the action potential and in modulating the membrane input resistance. Kim (2003) reviewed the main characteristics of the modulation of two-pore domain K^+^ channels by FFA (TREK-1, TREK-2, and TRAAK). These channels, activated by GPCRs, stretch, pH, and PUFA, are broadly expressed in the nervous system where they control neuronal excitability (Lee et al., 2011; Liu et al., 2014; Blin et al., 2016). The effect of PUFA on TREK channels has profound physiological implications, since TREK-1 was shown to mediate the neuroprotection induced by PUFA (Lauritzen et al., 2000; Heurteaux et al., 2004; Noël et al., 2011; Liu et al., 2014). There are certain structural features that FFA must necessarily satisfy to activate these K^+^ channels: the length of the carbonyl group, the unsaturation (saturated FFA are not effective) and the presence of the negatively charged carboxyl group (Patel et al., 2001; Kim, 2003; Noël et al., 2011). It has been demonstrated that the effect of PUFA on fatty acid-sensitive two-pore domain K^+^ channels does not require metabolic conversion of fatty acids into other bioactive molecules nor cytosolic messengers, enzymes or Ca^++^ (Horimoto et al., 1997; Maingret et al., 1999; Casavant et al., 2000; Lee et al., 2011). Thus, the effect of FFA could result from direct interaction with the channel protein, via partitioning into the lipid bilayer (e.g., causing changes in membrane fluidity), and/or involving membrane-delimited signal transduction mechanisms (Casavant et al., 2000). Compounds that mimic the effect of AA on membrane properties did not reproduce the effect of FFA on K^+^ channels (Patel et al., 2001). TREK and TRAAK are, however, mechano-gated channels that preferentially open by negative mechanical pressure (i.e., convex membrane curvature); it is thus possible that FFA are involved or even induce membrane alterations that lead to changes in membrane curvature (Patel et al., 2001). It has been reported that the cytoplasmic C-terminal region of near 30 amino acids located close to the 4th TM segment of the TREK-1 or TREK-2 K^+^ channels confers sensitivity to unsaturated FFA (Patel et al., 1998; Kim, 2003; Lee et al., 2011). Furthermore, using deletion analysis it has been reported that not only the C-terminal group but also the extracellular loop between the 1st TM domain and the 1st pore domain may be important for this effect (Patel et al., 2001).

*The large-conductance Ca*^++^*-activated K*^+^ *channels* (Slo1 BK channels), a very diverse group of channels resulting from alternative splicing in the CNS, are involved in the regulation of multiple physiological processes (Brenner et al., 2000). Similarly, a large number of cellular signaling molecules, including FFA, are known to modulate the function of Slo1 BK channels. For instance, BK channels are activated by FFA in GH_3_ cells (Denson et al., 2000), whereas a clonal line derived from GH_3_ (GH_4_C_1_) does not respond to AA, suggesting an alternative BK splice variant in these cells (Duerson et al., 1996). In GH_3_, BK channel activation by FFA requires a FA with at least one double bond, and a significant correlation was described between the degree of *cis*-unsaturation and the extent of channel activation (Denson et al., 2000). No significant correlation between changes in membrane fluidity and degree of activation was observed, and no effect of FFA metabolites was detected, leading to the conclusion that *cis*-unsaturated FFA interact directly with the channel protein proper. It was suggested that binding of the FFA to the Ca^2+^ binding site (a domain near the inner surface of the membrane in the cytosolic tail of the channel) could be responsible for changes of the channel protein conformation and hence changes in Ca^2+^ affinity, resulting in a conformation with a lower energy state (Denson et al., 2000). Further structural features of FFA required to increase BK channel activity have been defined: FFA must have a negatively charged head group and a sufficiently long (C > 8) carbon chain (Clarke et al., 2002). These authors showed that channel modulation is unlikely to be due to an alteration of the membrane electric field or the attraction of local counterions to the channel, concluding that FFA modulation of BK(Ca) channels could occur by direct interaction with either the channel protein or with some other channel-associated component. Sun et al. (2007) postulated a novel mechanism of β-subunit-dependent modulation of BK channels by AA. Further studies also suggested that docosahexanoic acid (DHA) directly activates the Slo1 channel complex with the auxiliary subunit β1 by destabilizing the closed conformation of the ion conduction gate (Hoshi et al., 2013a). Electrophysiological measurements showed that a single residue near the cytoplasmic end of S6 plays a critical role in the DHA activation of human Slo1 as no effect was observed on *Drosophila melanogaster* Slo1 channels, which normally do not contain DHA in the body (Hoshi et al., 2013b). Mutation of Y318, located probably at the cytoplasmic end of S6 in hSlo1 to Y318S, as found in dSlo1, cancels the effect of DHA on hSlo1 (Hoshi et al., 2013b).

One of the initial events in β-cells glucose stimulus-insulin secretion coupling is the closure of the *ATP-sensitive K*^+^ *channel*. It has been demonstrated that an increment in the concentration of FFA results in decreased insulin secretion (Corkey et al., 1989). The effect of FFA was shown to be dual: the initial products of phospholipase A2 digestion, or exogenous FFA, resulted in the reduction of ATP-sensitive K^+^ channel activity, whereas i) a long-term activation of these channels by AA, and ii) the reversal of this effect by AA metabolites via the cyclooxygenase pathway are also observed, possibly responding to a counter-regulatory mechanism (Eddlestone, 1995). Overnight incubation of clonal β-cells with palmitic acid induced a 50% decrease in the ability of glucose to stimulate insulin secretion and increased the pool of long-chain CoA (LC-CoA, the metabolically active form of FFA) (Larsson et al., 1996). Oleoyl-CoA increased the mean current 5-fold. Furthermore, the ability of LC-CoA to increase K^+^ conductance appears to be specific for the K_ATP_ channel (and not for the big conductance K^+^ channel, K_BK_, which is voltage and Ca^2+^-dependent, nor for 8-pS K^+^ channel). The stimulatory effect of the LC-CoA was dependent on both the acyl group (saturated or unsaturated chains with 14 to 18 carbons) and the CoA component. The accumulation of LC-CoA, possibly as a consequence of high glucose exposure (which inhibits fatty acid oxidation and elevates cytosolic LC-CoA levels), could be causally related to a loss of responsiveness to glucose. In tissue culture or non-insulin-dependent diabetes mellitus, long-term exposure to FFA impairs glucose-induced insulin by preventing the closure or promoting the opening of K_ATP_ channels (Larsson et al., 1996). LC-CoA esters with a chain length exceeding 12 carbons were also observed to be potent activators of the K_ATP_ channel in human pancreatic beta cells (Bränström et al., 2004). Previously it was demonstrated that fatty acid activation of ATP-sensitive K^+^ channels was most likely due to the participation of AA (and other *cis*-unsaturated fatty acid)-activated protein kinase C (PKC) isoenzymes and not by metabolites of AA via the cyclooxygenase or the lipoxygenase pathways (Müller et al., 1992). Similarly, a direct connection between AA and PKC activation was experimentally tested, explaining the mitogenic potential of AA (4-fold increase in DNA synthesis) in rat brown preadipocytes (Garcia et al., 2012). AA was in fact shown to activate PKC in various tissues, albeit by different mechanisms of action (Sekiguchi et al., 1987; Shinomura et al., 1991; Chen and Murakami, 1992; Blobe et al., 1995; Nishizuka, 1995; Nowicki et al., 1997; Leu et al., 2010). AA inhibition of Na^+^-K^+^-ATPase in sheep pulmonary artery was postulated to be mediated mainly by 20-HETE, the major metabolite of cytochrome P-450-arachidonic acid ω-hydroxylase pathway, through the activation of PKC (Singh et al., 2012). A similar mechanism was postulated in rat proximal convoluted tubules (Li et al., 2000). Nowicki et al. (1997) postulated that this inhibition was mediated by PKC-phosphorylation of Ser23 on the Na^+^, K^+^-ATPase α subunit. Another study suggested that 20-HETE can lead to PKC-dependent phosphorylation of Ser23 in Na^+^-K^+^-ATPase and of Ser896 in NMDA receptor NR1subunits at the putamen of piglets (Yang et al., 2012). A further study demonstrated that AA improved prostate cancer cell survival through 5-lipoxygenase (5-LOX) metabolites, a process which involved downstream PKCε activity (Sarveswaran et al., 2011). These authors further showed that treatment of prostate cancer cells with MK591, a 5-LOX inhibitor, or 5-LOX shRNA not only decreased PKCε expression but also diminished membrane localization of PKCε, inducing apoptosis. PKCε may thus be a mediator of survival signals downstream of 5-LOX metabolites (Sarveswaran et al., 2011).

Fatty acids act as substrates for acyl-coenzyme A (acyl-CoA) molecules by several synthases. These activated fatty acids can be short-chain (acetyl-CoA), medium-chain (e.g., octanoyl-CoA) or long-chain acyl-CoAs, such as palmitoyl-CoA. The effect of FFA and acyl-CoA esters on a highly active *plant* mitochondrial *ATP-sensitive K*^+^ *channel* (PmitoKATP) was studied in mitochondria isolated from durum wheat (Triticum durum Desf.) (Laus et al., 2011). Acyl-CoAs, linoleate and other FFA (laurate, palmitate, stearate, palmitoleate, oleate, arachidonate, and the non-physiological 1-undecanesulphonate and 5-phenylvalerate) directly activate PmitoKATP, but not through the Plant Uncoupling Protein (PUCP). The same activation effect was found to be widespread in mitochondria from different plant species and organs. Since PmitoKATP may act against environmental/oxidative stress (Atkin and Macherel, 2009), FFA activation in plants has been proposed to represent a physiological anti-stress mechanism: under hyperosmotic (NaCl or mannitol) stress conditions, FFA increase and may activate PmitoKATP strongly (Laus et al., 2011).

In rat atrial myocytes, perfusion of the cytoplasmic face of the membrane with unsaturated FFA (10–50 μM) such as AA, linoleic, and eicosatrienoic acids was reported to inhibit the ATP-sensitive K^+^ channel almost completely; lysophospholipids also markedly inhibited channel openings. In contrast, AA activated the *ATP-insensitive K*^+^ *channel* with an outwardly rectifying property. Since the FFA levels rise after long periods of ischemia, the authors speculated that the ATP-insensitive K^+^ channels contribute to a late phase of extracellular K^+^ accumulation (Kim and Duff, 1990).

*Ca*^++^*-dependent basolateral membrane K*^+^ *channel* (K_Ca_) activation in intestinal cells induces membrane hyperpolarization and a Cl^−^ secretory current. It was demonstrated that AA is a second messenger in this pathway: AA levels increase via Ca^2+^-dependent agonists through different pathways (PLA2, DAG, or DAG lipase) and induce a temporal modulation of Cl^−^ secretion. The inhibition of K_Ca_ can occur both through the extracellular or intracellular side of the channel and does not depend on the generation of either cyclooxygenase or lipooxygenase metabolites (Devor and Frizzell, 1998). This K_Ca_ inhibition was not specific for AA, as other FFA showed similar effects albeit to a lesser extent. A FFA effect caused by membrane fluidity changes was discarded as a plausible hypothesis, because different FFA act in a similar way on K_Ca_ but induce deviations from the optimal membrane fluidity in either direction (Devor and Frizzell, 1998). Hamilton et al. (2003) concluded that AA exerts a direct effect on K_Ca_ channel (identified as hIK1/hSK4) and described a second-messenger binding site for AA and other FFA. The AA sensitivity of hIK1 lies within the S5 pore and the S6 region, two amino acids (Thr^250^ and Val^275^) being crucial for this modulation. The side chains of both amino acids extend into the hIK1pore, suggesting that AA causes a direct pore block.

A medically important area of FFA modulation of ion channels is without doubt the mechanism underlying the anti-or pro-arrhythmic effects exerted on cardiac ion channels. PUFA were shown to reduce membrane electrical excitability of neonatal cardiac myocytes and provide an electrophysiological basis for the antiarrhythmic effects of these fatty acids (Kang et al., 1995). Research in this field has developed rapidly and insights into the action of FFA on cardiac myocytes have exploded. An example is the multiple and complex series of effects observed after *n*-3 supplementation, summarized by Moreno et al. (2012): “*n*-3 PUFA inhibit the fast sodium current (*I*_Na_), ultrafast activating delayed outward potassium current (*I*_Kur_), transient outward potassium current (*I*_to_), rapidly activating delayed rectifying outward potassium current (*I*_Kr_), L-type calcium inward current (*I*_Ca_), and NaC-Ca2C exchange current (*I*_NCX_), and enhanced slowly activating delayed rectifying outward potassium current (*I*_Ks_) and inward rectifying potassium current (*I*_K1_).” It is still not clear, however, which is the mechanism operative in n-3 FFA modulation of ion channels: is it a direct or an indirect effect? The prevalent opinion is to consider a direct interaction of FFA with the ion channel protein proper (Moreno et al., 2012 and references therein). Guizy et al. (2005) reported a blocking effect of both AA and DHA on human *ether-a-go-go*-related gene (HERG) channels, whose activation determines the duration of the action potential. This effect, which was found to depend on time, voltage and channel conformational state, was compatible with an open-channel block mechanism. The fact that ETYA caused a similar blockage effect suggests that these PUFA act through a direct mechanism and not through AA metabolism. Clearly, this mechanism requires the channel to be in an open state, but changes in the channel gating parameters suggest that these FFA also interact with the closed state. The inhibition action of these FFA on HERG channels can help to further explain the reported antiarrhythmic effects of AA and/or DHA (Guizy et al., 2005). Open-channel blockage by polyunsaturated FFA, apparently by binding of the FFA to an external site in the channel, was also reported for the major *voltage-dependent K*^+^ *channel (Kv1.5)* cloned from cardiac cells (Honoré et al., 1994; Guizy et al., 2008). In contrast to this effect, Gavrilova-Ruch et al. (2007) reported that AA activated human *ether à go-go* (hEAG) potassium channels expressed in CHO cells, an effect totally reversed upon washing with BSA. The potentiation effect was directly dependent on the number of *cis*-double bonds in the FFA, and probably involved a direct mechanism of action, as ETYA also potentiated hEAG currents, presumably acting through the outer membrane leaflet. The fact that FFA activate hEAG channels whereas they inactivate HERG channels may be due to structural differences in the pore mouth which, in the case of hEAG channels, would prevent the access of AA to the permeation pathway. These channels are normally expressed in neuronal tissue but also in various tumoral tissues, pointing to a possible oncogenic role; the activating effect of AA on EAG channels has therefore been related to an enhanced tumor proliferative rate (Gavrilova-Ruch et al., 2007).

### Anion channels

Anion channels, *ClC-2 Cl*^−^ *channels* in particular, are widely distributed in epithelial and non-epithelial tissues. One of the potentially lethal, inherited diseases affecting Cl^−^ channels is cystic fibrosis. The disease is caused by mutations in the gene encoding a cAMP-regulated, phosphorylation-gated Cl^−^ channel, the *cystic fibrosis transmembrane conductance regulator (CFTR*). CFTR-mediated Cl^−^ and bicarbonate transport drives fluid secretion across epithelial cells. In the gastrointestinal tract, the pathological alteration or loss of CFTR function severely hampers the production of exocrine pancreatic and intestinal secretions, leading to incomplete food digestion and malabsorption. In CF newborns, the defective fluid secretion may lead to a life-threatening obstruction of the distal small intestine, the so-called meconium ileus syndrome. In the pulmonary tract, the CFTR plays a major role in maintaining the airway surface protected by a fluid biofilm. It is therefore not surprising that the respiratory tract is often the target of secondary infections and pneumonia by the combination of CFTR and defective mucosal immunity, *Pseudomona aeruginosa* being the main pathogen involved. The CFTR is inhibited by several fatty acids in the following order: linoleic ≥ arachidonic ≥ oleic > elaidic ≥ palmitic ≥ myristic (Linsdell, 2000). The mechanism of AA inhibition has been suggested to result from the electrostatic interaction of the FFA with positively charged amino acids located at the cytoplasmic vestibule of the CFTR channel pore (Zhou and Linsdell, 2007), pointing to a more general inhibitory mechanism of apical membrane Cl^−^ channels by different FFA, which would act from the cytosolic surface thus rejecting the idea that inhibition results from changes in membrane fluidity (Anderson and Welsh, 1990).

Mutations in the CFTR Cl^−^ channel are associated with severe lung disease in about 5% of cystic fibrosis patients, a condition that may lead to death. Consequently, a possible therapeutic approach for cystic fibrosis is the potentiation of alternative pathways for Cl^−^ transport in the lung, including the targeting of ClC-2 Cl^−^ channels in the epithelium of the respiratory tract. These channels were found to be activated by oleic, elaidic and arachidonic acids and by cAMP-dependent PKA, but AA was found to increase the Cl^−^ currents in a PKC-and PKA-independent manner (Tewari et al., 2000). The increment of Cl^−^ currents was FFA dose-dependent, and only observed with unsaturated FFA indistinctly of *cis* or *trans* isomerism. The authors postulated that the effect of FFA may be due to direct effects on the channels, probably through mechanisms similar to those underlying the effect of FFA on the TREK-1 channel described above, and not to products of FFA metabolism (Tewari et al., 2000).

### Sodium (Na^+^) channels

*Cardiac Na*^+^ *channels* (the major class of ion channels that determines cardiac excitability) are also modulated by FFA, causing a reduction in the electrical excitability and/or automaticity of cardiac myocytes. Here again, it is postulated that the inhibition is dependent on FFA structure (Kang et al., 1995; Xiao et al., 1995), reversible in the presence of BSA, and not mediated by FFA metabolites (Kang and Leaf, 1996; Xiao et al., 1997). The mechanisms by which they exert their action remain uncertain; however, it is postulated that FFA act as non-competitive inhibitors, through a single class of sites, by an allosteric inhibitory mechanism (Kang and Leaf, 1996). These authors postulated a model in which the FFA hydrophobic portion interacts with the hydrophobic TM domains of the channel protein at either the lipid-channel interface or at the space between hydrophobic protein domains; the negatively charged carboxyl group interacts ionically with the positively charged amino acid residues of the channel protein near the surface of the bilayer where the carboxyl group is anchored. Cardiac Na^+^ channels consist of two subunits: the α subunit (the largest one, itself constituting a functional channel) and the β subunit (smaller; it interacts functionally with the regulatory segments of the Na^+^ channel) (An et al., 1998). FFA rapidly and strongly suppress voltage-gated Na^+^ currents in cells transfected with only the α-subunit of the human cardiac Na^+^ channel and prolong the duration of its inactive state, probably by binding to the inactivated form of the channel (in this state the channel displayed a 43-fold higher affinity for FFA than channels in the resting state) (Xiao et al., 1998). However, not only PUFA but all FFA exerted effects, leading these authors to the conclusion that the characteristic specificity of the effects of PUFA on native Na^+^ currents was lost in the exclusive presence of the Na^+^ channel α subunit. Xiao et al. (1998) also postulated that the configuration of the α-subunit may be more open or uncovered in the absence of other components of the intact voltage-dependent human cardiac Na^+^ channel, thus allowing even those FFA lacking the two or more double bonds to gain access to the site(s) at which PUFA affect conductance in the complete channel. The authors suggested that a short cytoplasmic segment of the transfected channel, linking the III and IV TM segments, was the site of action of FFA (Xiao et al., 1998). Co-expression of the β-subunit with the α-subunit of the human cardiac Na^+^ channel restores the selective effect of the PUFA, leading to the conclusion that the β-subunit modifies the FFA blockage of the Na^+^ channel (Xiao et al., 2000). A study with the hH1(α) Na^+^ channel led to the discovery of the importance of Asn 406 in the inhibition of cardiac voltage-gated Na^+^ currents by PUFA (Xiao et al., 2001). Another study using cells transfected with the *skeletal muscle sodium channels* (SkM1) isoform showed that ion channel modulation by FFA depends on the mode of FFA administration (Wieland et al., 1996). Intracellular AA exposure increased channel currents whereas the hH1 isoform did not show significant current increases. Thus, the response to FFA must include an isoform-specific element. In contrast, both isoforms were inhibited when unsaturated FFA were applied extracellularly. These results point to the existence of two distinct sites and mechanisms for FFA modulation of sodium channels: a potential extracellular site for extracellular FFA, and an intracellular one that appears to be exclusively for SkM1. The activation effect was observed over 120 min, suggesting that neo-synthesis and trafficking/insertion of new channels to/in the cell membrane could occur during this period, alone or in combination with unmasking of reserve channels already present in the plasmalemma (Wieland et al., 1996). This dual mechanism was also observed in the muscle *rNa(V)1.4* channel isoform, which appears to depend on the depolarizing potential: AA, but not its metabolites, increased the channel current evoked by a −30 or −40 mV depolarization of the membrane potential, but significantly decreased it by a depolarization over −10 mV (Gu et al., 2009). It was also reported that *cis*-unsaturated FFA with a double bond at position 9 have a biphasic effect on connexin 46 hemichannels: current activation at low FFA concentration and current inhibition at higher concentrations, the effect being directly proportional to the number of double bonds (Retamar et al., 2011). The authors ruled out the possibility that the biphasic effect was mediated by changes in the biophysical properties of the plasma membrane. They concluded that activation and inhibition current mechanisms involve different sites of action (Retamar et al., 2011).

A different dual mechanism of AA action has been postulated for the modulation of δ-opioid receptor (DOR) function (Sullivan et al., 2015). This modulation involves regulation by cyclooxygenase (COX) and lipoxygenase (LOX) dependent metabolites and activation of PKC. A COX-dependent metabolite of AA induces a responsive state of DOR that is capable of mediating antinociception and inhibition of adenylyl cyclase activity in response to opioid agonists, whereas a novel LOX-dependent metabolite of AA produces a loss of responsiveness of the DOR system. Sullivan et al. (2015) further demonstrated that exogenously added and endogenously produced AAs follow different metabolic processes, pointing to the existence of subcellular compartmentation of the enzymes involved. This dual regulation of DOR function may explain the observed variations in the efficacy of opioids in the treatment of pain. Another dual mechanism of FFA is described below for nicotinic acetylcholine receptors.

## FFA effects on ionotropic neurotransmitter ligand-gated ion channels

The regulation of rapid ligand-gated ion channels by fatty acids is illustrated using two paradigmatic cases, the γ-amino butyric acid receptor and the nicotinic acetylcholine receptor.

### γ-amino butyric acid receptor (GABA-R)

Exogenously added unsaturated FFA, or endogenously produced by the cell, modulate the GABA-R by drastically altering the binding characteristics of various GABA-R ligands, underlying the importance of the lipid environment for this process (Schwartz et al., 1988; Koenig and Martin, 1992; Samochocki and Strosznajder, 1993; Witt and Nielsen, 1994; Witt et al., 1996). FFA acting on the GABA-R must have at least one C-C double bond and a carbon length of 16–22 C (Witt and Nielsen, 1994). The effect of FFA on GABA/benzodiazepine receptor Cl^−^ channel complex from mammalian, avian, amphibian, and fish species was studied *in vitro* (Witt and Nielsen, 1994). Different effects of unsaturated FFA were observed on [^3^H]diazepam and [^3^H]muscimol binding: FFA enhanced ligand binding in the case of mammalian and amphibian receptors; of 17 fish species studied, 11 species presented weak stimulation of ligand binding, 4 species did not show augmented stimulation and 2 species exhibited inhibition; in the 10 bird species studied, only weak enhancement of [^3^H]muscimol binding was found, whereas [^3^H]diazepam binding was similar to mammal species (Witt and Nielsen, 1994). These results point to phylogenetic differences in the receptor that might account for the differences in FFA modulation. Again it is possible to consider a direct or an indirect FFA mechanism for GABA-R modulation. The argument for indirect effects rests on the fact that ontogenetic differences also involve changes in membrane composition, as fish membranes are composed mainly of phospholipids with unsaturated fatty acids and, hence, a further fluidizing effect by exogenously added unsaturated FFA is unlikely (Witt and Nielsen, 1994). However, an increase in the temperature of the binding assay, which induced an increment in membrane fluidity, did not alter the unsaturated FFA effect on ligand binding to GABA-R. Evidence pointing to a direct mechanism included the study of FFA effects on distinct recombinant human GABA_A_-R complexes formed by different subunit compositions (α, β, and γ subunits) where a modulation of the ligand binding by FFA was dependent on the subunit combination. These results suggest the existence of specific amino acid sequences in the α subunits that confer FFA sensitivity to the GABA_A_-R (Witt et al., 1996, 1999). Additional data suggest that FFA bind to specific sites, unleashing apparently independent responses: the rapid potentiation of the GABA currents and the increased desensitization of the GABA-R complex, which requires the presence of the γ2 subunit (Nabekura et al., 1998). Thus, the combination of different factors, such as the type and concentration of FFA plus the various combinations of GABA-R subunits in the same neuron result in a broad spectrum of potential modulatory mechanisms which might affect GABA responses in the CNS. The mechanism of FFA modulation on GABA_A_ receptors–activation of second messenger systems-appears to be shared by another inhibitory ligand-gated ion channel, the glycine receptor (Kloda et al., 2007).

### Nicotinic acetylcholine receptors (nAChRs)

“A protein isolated from Naja naja siamensis venom on the basis of its phospholipase A activity inhibits acetylcholine receptor function in post-synaptic membrane vesicles from *Torpedo californica*” (Andreasen and McNamee, 1977). This was the first evidence linking FFA with nAChR function. Shortly after, it was demonstrated that incorporation of unsaturated fatty acids or lyso-phosphatidylcholine into *Torpedo* membranes also inhibited nAChR function whereas lyso-phosphatidylethanolamine and most saturated FFA caused no effect (Andreasen et al., 1979). This inhibitory effect could be reversed and/or prevented by treatment with bovine serum albumin. Spin-labeled fatty acids also inhibited *Torpedo* nAChR, the magnitude of this effect being largely dependent on the position of the nitroxide group along the hydrocarbon chain (Andreasen and McNamee, 1980). The FFA inhibitory effect on nAChR function was attributed to the perturbation of protein-lipid interactions, and the magnitude of the effect was found to depend on FFA structure (Andreasen and McNamee, 1980). Alterations in nAChR function by FFA, particularly linolenic acid, or by phospholipase A2 hydrolysis products were directly correlated with perturbations of nAChR structure (Villar et al., 1988).

We analyzed the effect of four long-chain free fatty acids (AA, 20:4; DHA, 22:6; palmitic acid, 16:0; and nonadecanoic acid, 19:0) on the function of the acetylcholine receptor (nAChR) at the single-channel level (Bouzat and Barrantes, 1993a). The effect had a rapid onset and only very brief opening events were apparent after FFA application. The modification appeared not to be critically dependent on the degree of FFA saturation. In intact cells, fatty acids could reach and affect nAChR channels in the plasmalemma under the patch pipette when added from outside the patch-clamped area, suggesting i) that fatty acids diffused laterally and ii) the possibility that the AChR-lipid interface was the site of action of FFA (Bouzat and Barrantes, 1993a).

Free fatty acids (FFA) display the highest affinity for the native membrane-bound nAChR among all lipids studied to date (Marsh and Barrantes, 1978; Ellena et al., 1983; Dreger et al., 1997; Mantipragada et al., 2003). We subsequently disclosed the occurrence of independent sites for phospholipids and sterols in native nAChR membranes (Antollini and Barrantes, 1998), and found that these discrete sites were both accessible to FFA. From fluorescence quenching studies using nitroxide spin labels we also tentatively concluded that the sites were located at a shallow depth close to the phospholipid polar head region in native nAChR membranes (Barrantes et al., 2000). However, despite being located at the same site, each class of FFA differs in its effect on the physical properties of the membrane depending on its structure. Using the polarity-sensitive fluorescence probe Laurdan, it was possible to distinguish between saturated FFA, which induced a small increase in membrane order, and *cis*-unsaturated fatty acids, which caused a clear decrease of the lipid order. Double-bond isomerism could also be distinguished: oleic acid (18:1*cis*) induced a net disordering effect, whereas elaidic acid (18:1*trans*) produced no changes in membrane order (Antollini and Barrantes, 2002). These data lead us to suggest that it is the direct action of FFA at the lipid-protein interface, displacing essential lipids from their sites, rather than changes in bulk properties, such as membrane fluidity, that accounts for the inhibitory effect of FFA on nAChR function.

**Figure 1:**
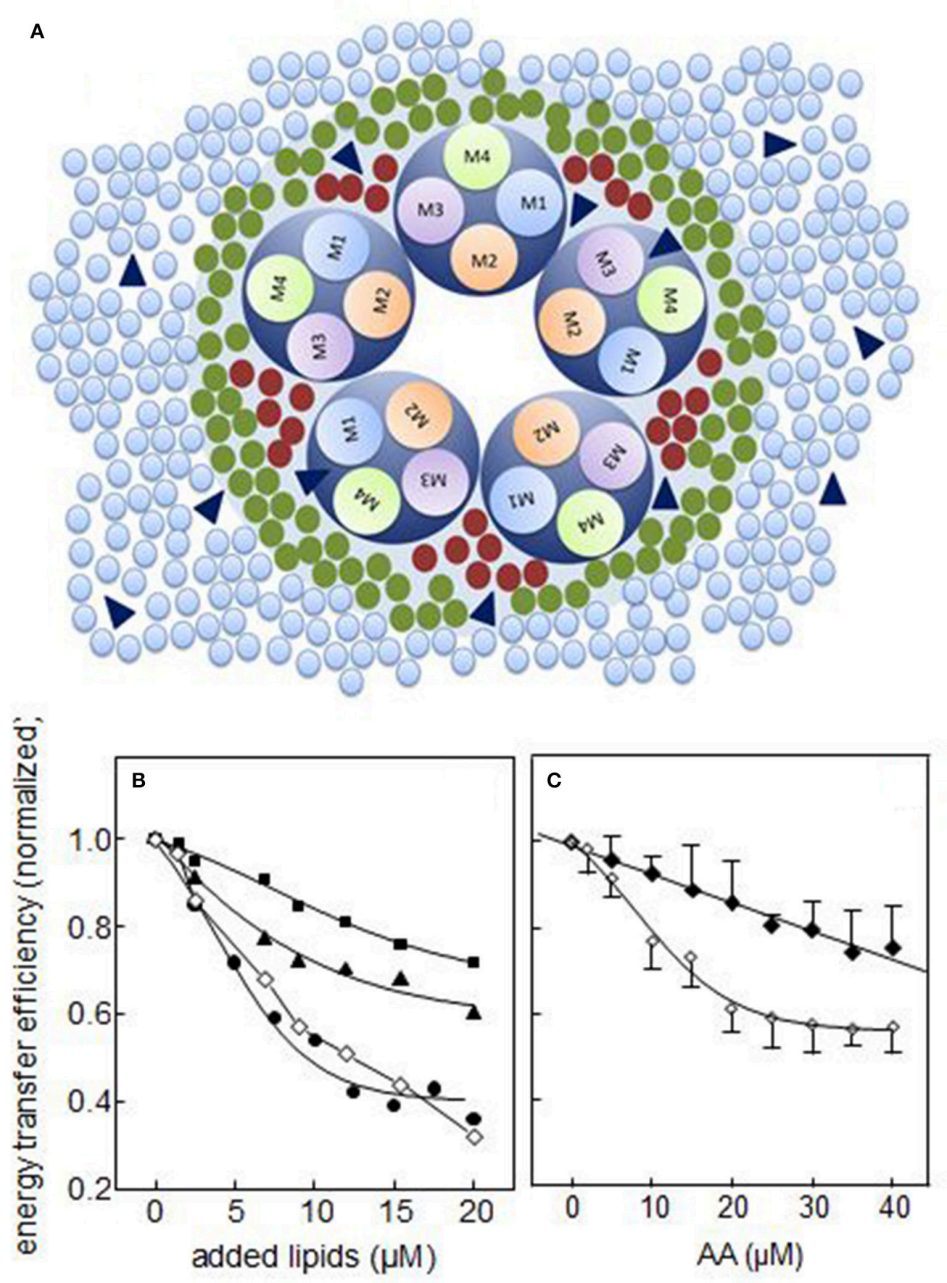
**(A)** Schematic diagram of a direct mechanism of action of FFA on nAChR function. The scheme illustrates the nAChR-lipid relationship in a receptor-rich membrane, outlining the spatial distribution of the transmembrane segments (TM1, TM2, TM3, and TM4) and the surrounding lipid shell. Three lipid topologies are indicated: non-annular lipids (bordeaux •), annular lipids (green •) and bulk lipids (light blue •). FFA (blue ▴) can be found in any of these three domains. **(B,C)** Experimental studies on annular and non-annular sites using the efficiency (*E*) of the Förster resonance energy transfer (FRET) process between the intrinsic fluorescence of *T. californica* nAChR membranes and the extrinsic fluorescent probe Laurdan; **(B)** in the presence of increasing concentrations of DOPC (■), cholesterol hemisuccinate (CHS, ▴), and oleic acid (•), where the symbol (♢) corresponds to the sum of *E* of DOPC and CHS (from Antollini and Barrantes, 1998) and **(C)** in the presence of increasing concentrations of arachidonic acid with the nAChR in the resting (“R”, ♢) or the desensitized (“D”, ♦) state. The latter was generated by incubation of the membrane with 1 mM carbamoylcholine prior to the fluorescence measurements (From Fernández Nievas et al., 2007).

Two types of lipid sites have been described to be present in integral membrane proteins in general, and at the lipid-nAChR interface in particular: annular and non-annular sites. Annular sites constitute the first shell of lipids surrounding the protein and interact with the protein in a relatively less specific manner; the rate of exchange between annular shell lipid and bulk membrane lipid is relatively fast, in the order of 1–5 × 10^8^ s^−1^ (Marsh and Barrantes, 1978; Barrantes, 2004). Non-annular lipid sites involve spaces between TM helices and between subunits in multisubunit proteins (Lee, 2004). Non-annular lipids are considered essential for protein activity, and display higher specificity for the protein. The rate of exchange of non-annular lipids with bulk lipids has not yet been experimentally determined, but is presumably sluggish, and in any case much slower than that of annular lipids, as a result of the high specificity of the interaction between non-annular lipids and the protein (Lee, 2004). On the basis of competition studies, early studies suggested that non-annular lipids were associated with binding sites to which cholesterol is bound but phospholipids are not (Jones and McNamee, 1988). We demonstrated that both endogenous FFA generated by phospholipase A2 from *Torpedo* native membranes and AA exogenously added to these membranes localize at both annular and non-annular sites at the lipid-protein interface (Fernández Nievas et al., 2007). Furthermore, we found that nAChR conformational transitions between the resting (R) state and the desensitized (D) state may entail a rearrangement of the nAChR TM region involving the occlusion of non-annular sites at the lipid-protein interface or simply decreased lipid efficacy in accessing such sites (Fernández Nievas et al., 2007; Figure 1).

**Figure 2:**
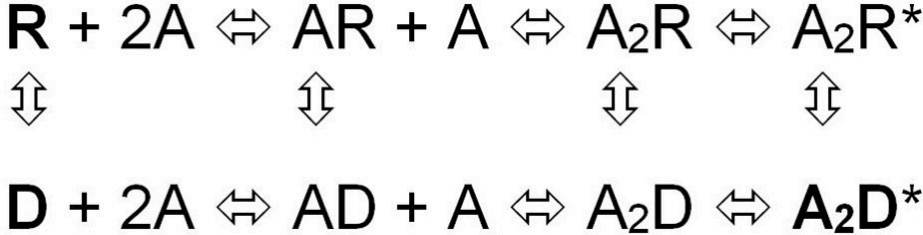
**Sequential model of the nAChR activation equilibrium**. The model considers that each state of channel activation has a corresponding desensitized state (Dilger and Liu, 1992): R is the nAChR in the resting state, A the agonist and RA and RA2 represent the nAChR with one or two agonist molecules bound, respectively, and RA2^*^ is the biliganded nAChR in a transient active open configuration; D, AD, A2D, and A2D^*^ are the corresponding isoforms in the desensitized, non-conductive states, respectively. This allosteric equilibrium can be affected e.g., by single-point mutations and exposure to some drugs (From Fernández Nievas et al., 2008).

Taking advantage of the different affinities that the fluorescence dye crystal violet (CrV) displays for the resting (R) and desensitized (D) states of the nAChR we observed that the dissimilar effects that FFA exert on the receptor conformational states depend on the structural characteristics of the fatty acids (Fernández Nievas et al., 2008). Whereas *cis*-FFA increased membrane polarity, *trans*-FFA and saturated FFA caused essentially no changes in this property. Only *cis*-FFA drove the nAChR out of the R state in the absence of agonist; we hypothesized that most likely direct contacts between the FFA and TM portions of the nAChR are responsible for driving the receptor out of the R state and, hence, inhibit its function. *cis*-FFA caused a second effect on membrane-bound nAChRs: they prevented the receptors from reaching the D state in the presence of agonist. This lack of transition to the D state of the nAChR could be mimicked by raising the temperature, which disorders the membrane bilayer, or by treatment with PLA2, which decreases the polarity of the membrane, two conditions that do not perturb the R state of the nAChR. The second effect seems not to depend on the presence of specific molecules at the lipid-nAChR interface but rather to be unspecific, mainly associated with changes in the physical state of the bulk membrane. Thus, whereas foreign molecules at the lipid-protein interface probably modify the “activation gate” of the nAChR-associated channel, leading to an intermediate D state, changes in the physical state of the membrane (particularly changes in membrane order or polarity) are likely to perturb the “desensitization gate” (Fernández Nievas et al., 2008; Figure 2). A previous study with single cells obtained from flexor digitorum brevis muscles of adult male mice also correlated nAChR functional inhibition with nAChR protein conformation (Nojima et al., 2000). These authors demonstrated that AA, and prostaglandin D2 (PGD2) or its metabolites, cooperatively accelerate desensitization of the nAChR. The explanation provided invoked the activation of PKC by AA and PGD2 in this mechanism. However, the experiments of Fernández Nievas et al. (2008), performed in isolated nAChR-rich plasma membranes in the absence of phosphorylation (absence of ATP and insufficient Mg^2+^ concentration; Safran et al., 1990) clearly demonstrated the inhibition and conformational changes of the nAChR, suggesting that the inhibition and desensitization of the nAChR by FFA does not involve PKC activity. Putting all the information together reinforces the view that there is more than one mechanism of action involved in the modulation of nAChR function by FFA.

In order to further dissect the mechanisms of action of *cis*-unsaturated FFA on the native membrane-bound *Torpedo* nAChR, we resorted to the use of five different monounsaturated fatty acids with the same number of carbon atoms (*cis*-6-18:1, *cis*-9-18:1, *cis*-11-18:1, *cis*-13-18:1, and *trans*-9-18:1) (Perillo et al., 2012). Four out of five 18:1 FFA tested (*cis*-9-18:1, *cis*-11-18:1, *cis*-13-18:1, and *trans*-9-18:1) were found to localize at both annular and non-annular sites; only *cis*-6-18:1 was found in annular sites. Membrane order was found to undergo a very slight and saturable decrease in the presence of the *trans*-unsaturated FFA, whereas all *cis*-monounsaturated FFA tested caused membrane order perturbations dependent on the position of the double bond. The largest effect was observed when the double bond was near the middle of the acyl chain. Patch-clamp experiments disclosed the inhibition of nAChR currents only with *cis*-9-18:1 or *cis*-6-18:1. All unsaturated FFA prevented the nAChR from reaching the D state in the presence of agonist but only *cis*-9-18:1, *cis*-11-18:1, and *cis*-13-18:1 drove the AChR out of the R state in the absence of agonist. Finally, only *cis*-monounsaturated FFA caused a local topological change in the nAChR γTM4 segment (*cis*-6-18:1 to a lesser extent) (Table 1). Taken together, these data led us to conclude that the position of the torsion angle of unsaturated FFA is a key factor in channel blockage. If one takes into consideration that (i) the sequence of structural events coupling ligand binding to channel gating begins with movements at the ligand-binding loops, is followed by the displacement of loops located at the interface between the extracellular ligand binding domain and the TM domain, the subsequent tilting/bending of the pore-lining M2 helix, and ends with movements of M4, M3, and M1 helices in the TM domain (Mitra et al., 2004; Auerbach, 2005), and (ii) that FFA might exert their action by an allosteric mechanism at the lipid-nAChR protein interface, those FFA with the double bond at a shallow position in the membrane probably share the topological loci of the conserved core structure for nAChR gating and hence could perturb this synchronous mechanism (Perillo et al., 2012).

**Table 1:** **Summary of effects produced by different FFAs on the nAChR (Perillo et al., 2012)**.

	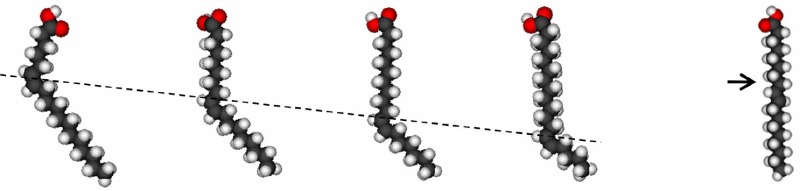				
	Petroselenic acid (18:1 *cis*-**6**)	Oleic acid (18:1 *cis*-**9**)	Vaccenic acid (18:1 *cis*-**11**)	Octadecanoic acid (18:1 *cis*-**13**)	Elaidic acid (18:1 *trans*-**9**)
Sites at lipid-nAChR interface	A^a^	A + NA^b^	A + NA	A + NA	A + NA
inhibition of nAChR function	+	++	–	–	–
GP modification	++	+++	++	+	–
Anisotropy modification	+	+++	++	++	–
nAChR TM4 perturbation	+	++	++	++	–
R-state perturbation	–	++	++	++	–
D-state perturbation	++	++	+	+	+

*The CPK structures of the FFAs studied are shown for clarity: the broken line shows the position of the cis double bond and the arrow indicates the position of the trans double bond*.

a A, annular sites;

b *NA, non-annular sites*.

Changes in membrane lipid composition (Criado et al., 1984; Sunshine and McNamee, 1994; Baenziger et al., 2000; da Costa et al., 2002) and/or the presence of exogenous hydrophobic molecules like steroids and fatty acids (Andreasen and McNamee, 1980; Villar et al., 1988; Bouzat and Barrantes, 1993a,b, 1996; Lasalde et al., 1995; Nurowska and Ruzzier, 1996; Santiago et al., 2001; Garbus et al., 2001, 2002) affect AChR function. Individual amino acid mutations in the lipid-facing outer ring (M4) of the nAChR TM domain also modulate nAChR function (Lee et al., 1994; Lasalde et al., 1996; Ortiz-Miranda et al., 1997; Bouzat et al., 1998; Tamamizu et al., 1999, 2000), a clear indication that although far away from the agonist sites and from the ion channel, the M4 TM segment, the only member of the outermost TM ring (Barrantes, 2003), effectively influences nAChR function. In this sense, M4 would behave as a sensor of the lipid-protein environment unleashing a signal to the M2 channel region, probably initiated by the induction of changes in the topology of the outer ring and ultimately causing a conformational change of the whole AChR (Xu et al., 2005; Fernández Nievas et al., 2007, 2008; Perillo et al., 2012; Barrantes, 2015).

Contrary to the rapid inhibitory effect described above, a potentiation effect of AA on *Torpedo* nAChR expressed in *Xenopus* oocytes, lasting over 30 min after FFA washing, has been reported (Ikeuchi et al., 1996). This effect resembles the dual action of FFA on Na^+^ channels described in previous sections. Pretreatment with PKC inhibitors did not counteract the FFA inhibition but abolished the potentiation effect. However, experiments performed with mutant nAChR lacking PKC phosphorylation sites showed no current blocking effect of AA and a current potentiation greater than in the control condition. Together, these results pointed to two different and independent mechanisms: a short-term blocking mechanism through direct action of AA on nAChR PKC phosphorylation sites, rather than through an effect on the membrane environment; and a long-term potentiating mechanism caused by PKC activation not involving nAChR phosphorylation. This second mechanism must include an unknown pathway related to AA-regulated PKC. Different results were observed depending on the FFA structure. Treatment with linoleic (18:2) or linolenic (18:3) acids caused similar dual effects on *Torpedo* nAChR; however, the long-term potentiation was not evident in mutant nAChR, indicating that these FFA, in contrast to AA, did in fact potentiate nAChR via its PKC phosphorylation site (Nishizaki et al., 1997a). On the other hand, current potentiation by treatment with oleic acid (18:1) was not hindered by PKC inhibitors but fully inhibited by KN-62, a calmodulin-dependent protein kinase II (CaMKII) inhibitor, suggesting that oleic acid enhances nAChR currents by activation of CaMKII, independently of the PKC pathway (Nishizaki et al., 1997b). This can be accounted for by the explanation provided by Gérczy et al. (2012), pointing to the AA selectivity of certain PKC isoforms over several other signal-transduction mechanisms. Lysophosphatic acid also enhances *Torpedo* nAChR currents both in control and in mutant nAChRs lacking PKC phosphorylation sites; however, the potentiation was also completely blocked by GF109203X (Nishizaki and Sumikawa, 1997). These authors suggested that lysoPA probably potentiates nAChR currents by pertussis toxin-insensitive G-protein and activation of Ca^2+^-dependent/-independent PKCs with subsequent phosphorylation of the receptors and, additionally, by an unknown factor or process activated by PKC activation. Saturated FFA with less than 20 C atoms also potentiate nAChR currents with the participation of PKC, stearic acid (18:0) being the most potent FFA in this respect (Ohta et al., 2003). A comprehensive study of the importance of the FFA structure (Yaguchi et al., 2005) concluded that FFA modulation of nAChR current could not be simply explained by the number and position of the *cis*-double bonds. This study showed that 20:1ω12 (**8**-eicosenoic acid) potentiated the currents without depression, whereas 20:1ω15 (**5**-eicosenoic acid) and 20:1ω9 (11-eicosenoic acid) elicited only the depression component, suggesting that *cis*-double bond at the 8th position plays a role in the potentiation and at the 5th or 11th position in the depression. This rule agrees with the fact that 20:3ω9 (5,8,11-eicosatrienoic acid), 20:4ω6 (5,8,11,14-eicosatetraenoic acid), and C20:5ω3 (5,8,11,14,17-eicosapentaenoic acid) induced a transient inhibition followed by enhancement of the currents, whereas 20:3ω6 (8,11,14-eicosatrienoic acid) induced only nAChR depression and not potentiation (Yaguchi et al., 2005). It should be noted, however, that 20:2ω6 (11,14-eicosadienoic acid) has no double-bonds at crucial positions and it has a dual effect on nAChR currents. The carboxy-terminal group appears to play a critical role in the potentiation of the nAChR, as this group is necessary for PKC-ε activation; replacement of COOH in linoleic acid by CONH2 (linoleoylamide) only caused the depression effect (Yaguchi et al., 2005).

The work of Vijayaraghavan et al. (1995) was the first demonstration that FFA also modulate neuronal-type nAChRs. Using chick ciliary ganglion neurons these authors showed that seconds of incubation with AA sufficed to inhibit neuronal nAChR with a distinct and largest effect on the α7-type nAChR. This inhibition was not restricted to AA but also included other FFA in a structure-dependent manner: saturated or cis/trans monounsaturated FFA showed little or no effect; FFA having two or three double bonds exhibited inhibitory effects, and AA displayed maximal effect. The authors concluded that the inhibition must be caused by a direct action of the FFA on the nAChR or through the membrane, discarding the possible action of AA metabolites or the activation of PKC. Minota and Watanabe (1997) also showed that AA directly inhibits nAChR in bullfrog sympathetic ganglia, and that its metabolites do not play a major part in this inhibition. Two general possibilities were postulated to account for this mechanism: (i) AA binds to allosteric sites of the nAChR –one possibility being a site in the channel pore- thus inhibiting synaptic transmission without affecting the binding of ACh to the receptor; or (ii) AA perturbs the local environment of the receptors by partitioning into the membrane and thereby indirectly inhibiting receptor function. Nishizaki et al. (1998) indicated that neuronal nAChRs display short-term depression and/or long-term enhancement of nAChR currents depending on the biological source of the neuronal nAChR. Treatment of chick α7 nAChR with AA caused only a depression effect (in accordance with Vijayaraghavan et al. (1995)) whereas in the case of rat α7 nAChR the same treatment caused exclusively a potentiation effect by PKC activation. This strongly suggests a relation between FFA effect and nAChR structure; the different response to AA may probably be due to structural differences between receptors. Further studies with rat α7 and α2β4 nAChR showed that AA increases glutamate release by potentiating the activity of presynaptic nAChRs, predominantly α7 nAChR, under the influence of PKC, an effect not related to CaMKII activity (Nishizaki et al., 1999). Stearic acid has also been shown to potentiate α7 nAChR currents, with the involvement of PKC in this potentiation (Ohta et al., 2003). The latter work demonstrated that although stearic acid enhances activity of already active PKC-ε it does not directly activate this enzyme.

Studies of the FFA modulation of different proteins through PKC indicate that, in some cases, the mechanism involves not a direct PKC activation but an increased expression and altered subcellular distribution of PKC. It is known that translocation of PKC isoforms is generally regarded to be indicative of their activation. Li et al. (2010) explained the induced pulmonary artery (PA) contraction by hypoxia through an important increment in both the RNA levels and protein expression of PKC-δ and PKC-ε induced by 15-hydroxyeicosatetraenoic acid (HETE). In this case, AA is metabolized by 15-lipoxygenase, which is up-regulated by hypoxia, to 15-HETE which causes pulmonary artery (PA) constriction by activation of PKC-δ and PKC-ε. A similar profile was described in HEK293 cells, where AA displays a biphasic effect on Ca^2+^ signaling: at low concentrations, AA suppresses both Ca^2+^ release and Ca^2+^ influx responses to agonist; whereas at high concentrations, AA potentiates Ca^2+^ release and Ca^2+^ entry response (Chen et al., 2012). These authors postulated that AA induces PKCα and PKCβII redistribution at the plasma membrane, and that at higher AA concentrations trafficking of PKCβI and PKCβII to the endoplasmic reticulum also occurred. A totally opposite effect was described in a study of the immunomodulatory properties of PUFA in phagocyte function (Gorgani et al., 2011). These authors demonstrated that AA causes a significant decrease in CRIg expression at both the mRNA and protein levels, and that this down-regulation is dependent on PKC activation by AA excluding the possibility that AA could exert its effect through its metabolism via cyclooxygenase and/or lipoxygenase.

It is important to highlight that the FFA concentrations used in all the studies mentioned here were below the high FFA levels reported in several pathological conditions as a consequence of PLA2 activation and FFA release from plasma membranes. Arachidonate levels in the plasma of malaria patients are in the order of 100 μM (≈ 33.3 μg·mL^−1^) (Eissen, 1993) and under ischemic conditions these levels can rise to 500 μM, 10-fold higher than the free AA levels found in normal brain (Yasuda et al., 1985). A 10-fold increment in FFA was also reported in an experimental model of acute lung injury (Arbibe et al., 1998).

**Figure 3:**
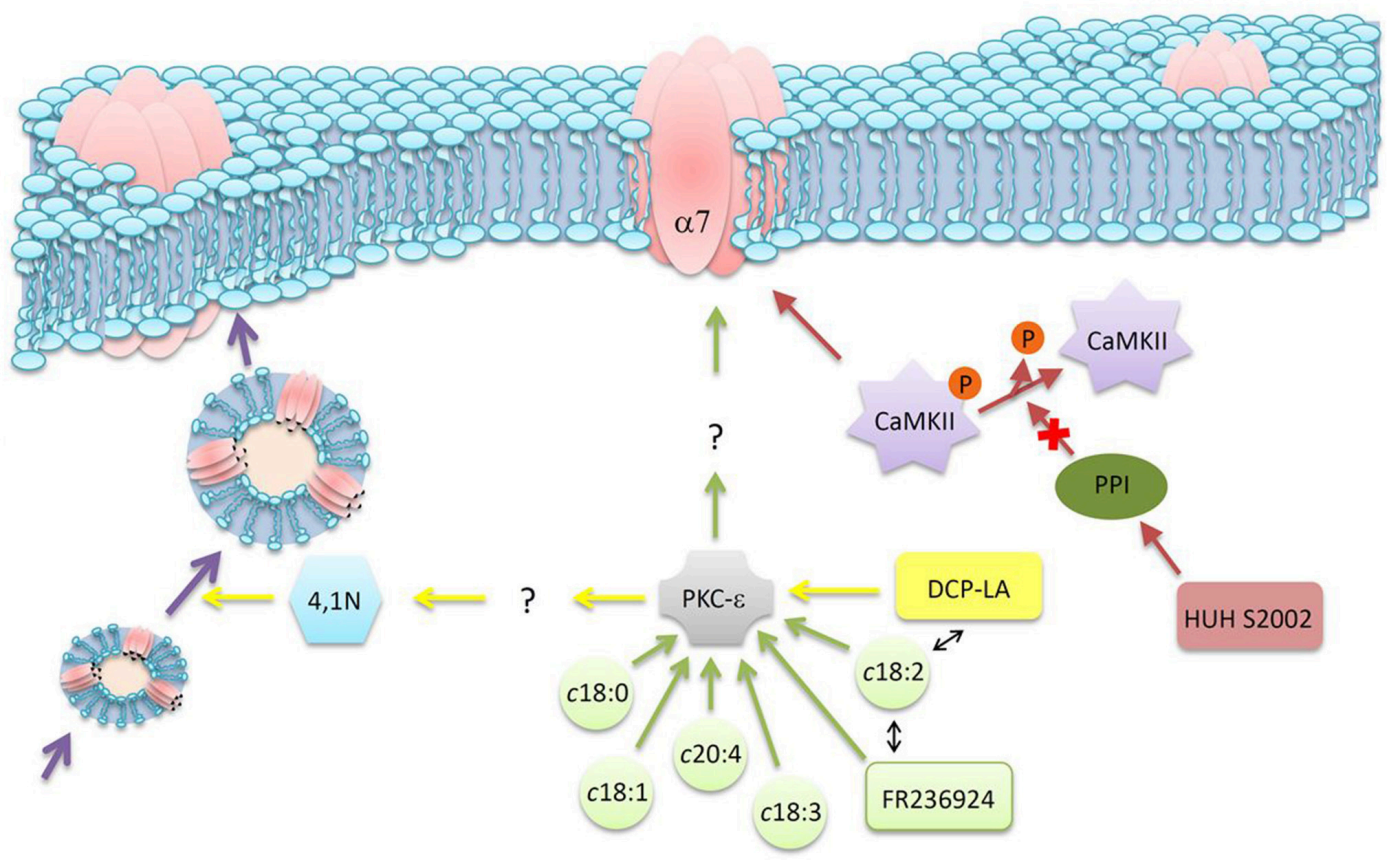
**Schematic diagram of indirect mechanisms of action of FFA on neuronal nAChR function**. Different FFA, or their derivatives, can modulate α7 nAChR through various signaling pathways. See text for details.

More recent studies used FA derivatives to explore in more detail the modulatory mechanism exerted by FFA on α7 nAChR. FR236924, a linoleic acid derivative, was synthesized having cyclopropane rings instead of *cis*-double bonds. The FA analog induced a long-lasting facilitation of hippocampal neurotransmission, as assessed by the persistent enhancement in the activity of presynaptic nAChRs via a PKC pathway (Tanaka and Nishizaki, 2003; Yamamoto et al., 2005). 4-[4-(Z)-hept-1-enyl-phenoxy] butyric acid (HUHS2002) potentiated rat α7 nAChR currents, an effect that was not affected by the addition of an inhibitor of PKC but significantly inhibited by an inhibitor of CaMKII (Kanno et al., 2012a). This suggests that HUHS2002 potentiates α7 nAChR currents by activation of CaMKII. However, HUHS2002 might indirectly activate CaMKII by inhibiting protein phosphatase 1 (PP1), which normally dephosphorylates and inactivates CaMKII. Another linoleic acid derivative with cyclopropane rings instead of *cis*-double bonds (DCP-LA) preserved the potentiation effect on nAChR currents, which was abolished by GF109203X, a PKC inhibitor (Kanno et al., 2012b). The DCP-LA effect (a) was significantly inhibited by vesicular transport inhibitors, (b) promoted the translocation of the α7 nAChR from the cytosol to the plasma membrane and (c) stimulated α7 nAChR delivery toward presynaptic terminals. The evidence suggests that the nAChR current potentiation could arise from the DCP-LA mediated stimulation of nAChR vesicular transport and the consequent increase in the number of nAChR targeted to the cell surface (Kanno et al., 2012b). This was the first postulation of PKC control of intracellular α7 nAChR trafficking; which PKC targets participate in this regulation is still not known. Subsequent studies showed that DCP-LA significantly increased an association of 4.1N -a scaffolding protein- with α7 nAChR; and that this association is partially prevented by GF109203X, an inhibitor of PKC, but independently of 4.1N phosphorylation (Kanno et al., 2013). The same study showed for the first time that 4.1N is required for translocation of the α7 nAChR toward the plasma membrane. Thus, DCP-LA could induce an increase in the association of the α7 nAChR with 4.1N in a PKC-dependent manner, not caused by PKC phosphorylation of 4.1N or by phosphorylation of the receptor. Probably DCP-LA, by activating PKC, phosphorylates an unknown factor that enhances the association of 4.1N with the α7 nAChR (Kanno et al., 2013). Thus, the evidence described in the last paragraphs point to a novel pathway linking lipid signaling to α7 nAChR responses, as is graphically summarized in Figure 3.

One avenue that needs to be explored in greater detail is the involvement of abnormalities of FA metabolism in brain diseases. Circulating long-chain fatty acids act as signals of nutrient surplus in the hypothalamus. Furthermore, pharmacological and/or genetic inhibition of FA synthase, AMP-activated protein kinase and carnitine palmitoyl transferase 1 (CPT1) results in marked decreases in feeding levels and loss of body weight in rodents (López et al., 2007). CPT1c, the recently discovered brain isoform of the multiprotein complex enzyme carnitine palmitoyl transferase, is predominantly localized in regions involved in the regulation of food intake, such as the hypothalamus, emotion and reward systems -the amygdala- and learning and memory, the hippocampus. CPT activity has been associated with dysregulation of insulin equilibrium in brain and related metabolic dysfunctions, and implicated in the evolution of Parkinson's and Alzheimer's diseases (Virmani et al., 2015).

## Conclusions

When analyzing the effects of FFA on a variety of ion channels, one of the first clear outcomes is that different FFA exert distinct effects, or no effect at all, on a same channel protein, whereas similar FFA may cause diverse effects on different proteins, even if the latter are very closely related from a phylogenetic point of view. In spite of these apparent discrepancies, it is clear that almost all FFA that modulate the ion channel directly, i.e., not through their metabolites or signaling cascades, act *by direct physical contact with the protein*. Direct mechanisms consequently put both FFA structure and channel structure -particularly those amino acids that participate in the interaction-at center stage. Amino acid residues involved in the recognition of FFA are apparently sensitive to the length, isomerism and saturation of the FFA. It is thus mandatory to identify and characterize in detail the structure of the intervening binding site(s) in the protein and the counteracting FFA structure to unravel the different inhibitory or stimulatory modalities at the molecular level and eventually contribute to the design of new lipid-based modulatory drugs targeting specific channel proteins.

## Author contributions

Experimental work quoted in this review was supported by grants PICT 2011-0604 from FONCYT, Ministry of Science and Technology and PIP No. N° 112-201101-01023 from the National Scientific and Technical Research Council of Argentina (CONICET) to FJB. and grants PIP 112-201101-00239 from CONICET, PGI 24/B217 from Universidad Nacional del Sur, and PICT 2012-2746 from MINCYT to SSA. The costs of publication were defrayed from grant PIP No. 112-201101-01023 from CONICET to FJB.

### Conflict of interest statement

The authors declare that the research was conducted in the absence of any commercial or financial relationships that could be construed as a potential conflict of interest.

## Abbreviations

FFA: free fatty acids
nAChR: nicotinic acetylcholine receptor
PUFA: polyunsaturated fatty acids
TM: transmembrane
VLCFA: very long-chain fatty acids.
